# Nearly Complete Genome Sequence of a Serotype 1 Fowl Adenovirus Strain Isolated in Jiangsu, China

**DOI:** 10.1128/MRA.00310-19

**Published:** 2019-07-25

**Authors:** Mengdi Zhang, Yage Hu, Chengcheng Zhang, Mengjiao Guo, Yongzhong Cao, Xiaorong Zhang, Yantao Wu

**Affiliations:** a College of Veterinary Medicine, Yangzhou University, Yangzhou, Jiangsu, China; b Joint International Research Laboratory of Agriculture & Agri-Product Safety, Yangzhou University (JIRLAAPS), Yangzhou, Jiangsu, China; KU Leuven

## Abstract

Here, we report the nearly complete genome sequence of nonpathogenic serotype 1 fowl adenovirus (FAdV) strain JS2017, which was isolated in Jiangsu Province of China. The JS2017 genome is 43,681 bp long. We propose that this virus could serve as a viral vector for future poultry vaccine research.

## ANNOUNCEMENT

Fowl adenoviruses (FAdV) are members of the genus Aviadenovirus, family Adenoviridae, and currently comprise subgroups I, II, and III. In contrast with the clear association of subgroup II and subgroup III FAdV with disease, the role of most subgroup I FAdV as pathogens is not well defined (1). Subgroup I FAdV can be subdivided into five genotypes (A to E) and 12 serotypes (FAdV-1 to FAdV-8a and FAdV-8b to FAdV-11) (2, 3). FAdV-1, FAdV-2, FAdV-8a, FAdV-8b, and FAdV-11 can cause inclusion body hepatitis (4, 5), and FAdV-4 can result in hydropericardium syndrome (6).

We collected livers from chickens with mild respiratory symptoms in Jiangsu Province of China, and liver homogenate was inoculated into 9-day-old specific-pathogen-free (SPF) chicken eggs via the chorioallantoic membrane. Virus JS2017 was further propagated on a chicken liver cancer cell line (LMH) for 72 h. Viral double-stranded DNA (dsDNA) was extracted from cell culture using the phenol-chloroform method. The hexon (including the loop 1 [L1] hexon region) was amplified and sequenced using Sanger sequencing to identify the group and type of FAdV (7). The BLAST search results showed that the isolate had 98.25% to 99.23% similarity to other FAdV-1 strains. To better understand the genetic characteristics of FAdV-1, the nearly complete genome sequence of strain JS2017 was amplified by PCR using 34 overlapping pairs of primers (Table 1). All PCRs were performed according the instructions for *TransTaq* HiFi DNA polymerase (TransGen Biotech). The 34 overlapping PCR products were cloned and Sanger sequenced (General Biosystems, Anhui, China). Each base was covered by three individual Sanger reads. Vector sequences were removed by the EditSeq program (Lasergene 8.0), and the full-length sequence was assembled using SeqMan (Lasergene 8.0). The nearly complete genome of JS2017 is 43,681 nucleotides (nt) long, with a 54.29% G+C content. Thirty-four open reading frames (ORFs) were predicted within the genome using the ORFfinder tool (https://www.ncbi.nlm.nih.gov/orffinder/), and the inverted terminal repeat (ITR) sequences are 54 nt long. A comparison of the nucleotide identity of JS2017 with four FAdV-1 reference strains, CELO (GenBank accession no. U46933), W-15 (KX247011), 61/11z (KX247012), and JM1-1 (MF168407), showed similarities of 99.3%, 99.6%, 99.7%, and 99.3%, respectively. The JS2017 strain possessed multiple insertions and deletions compared to reference FAdV-1 strain CELO, including in ORF8, ORF14, and the IVa2 and L4 100K genes. Amino acid comparison showed that the major hexon capsid protein of JS2017 has high similarity, ranging between 99.6% and 100%, with those of these four FAdV-1 strains. Furthermore, compared with those of reference strain CELO, the penton and fiber-2 of JS2017 have four and six amino acid mutations, respectively.

**TABLE 1:** Primers used to amplify the complete genomic sequence of FAdV-1 strain JS2017

Nucleotide position	Primer name	Sequence (5′→3′)
18587-19377	Hex-F1	GAYRGYHGGRTNBTGGAYATGGG
	Hex-R1	TACTTATCNACRGCYTGRTTCCA
1–1414	FAdV-1-1-F	GATGATGTATAATAACCTCAAAAACTAAC
	FAdV-1-1-R	CAGACAATGTCCGTATGTGTCG
1329–2784	FAdV-1-2-F	GATAGGACCCTTGCGACGA
	FAdV-1-2-R	CTCCACGCCACAAACTGTTT
2623–4139	FAdV-1-3-F	CAGCCAGGCGGATATTTGTA
	FAdV-1-3-R	CATCAGATGATGGAAGCCGA
4020–5444	FAdV-1-4-F	CTTGGCGTTGGAAAGTCTTC
	FAdV-1-4-R	CCCCAAACTAACCGATCTCG
5360–6792	FAdV-1-5-F	CGGTTCCTGAGTCTCTTAACG
	FAdV-1-5-R	CATTCACAACGAGCAGCTCA
6689–8171	FAdV-1-6-F	GATTTGGGTGCTCATTGTCG
	FAdV-1-6-R	GAAATGTTAGACCCACTCCCTC
8066–9512	FAdV-1-7-F	TGAGGATATCTATGACGGTGACC
	FAdV-1-7-R	GACGAAGGCTTCTATTGGCTA
9402–10786	FAdV-1-8-F	GTAGCGGTCGACTAGATTTTGG
	FAdV-1-8-R	CCGAACATATCGCCTCTACAC
10625–12018	FAdV-1-9-F	GTTGGATTGCTCGCTCCATA
	FAdV-1-9-R	CCGTTTAATGCAACTCCGAG
11879–13343	FAdV-1-10-F	GGTTGTCGAAAAGGTAATCGTT
	FAdV-1-10-R	CAGATGAAGTCATCCTCGTCG
13168–14591	FAdV-1-11-F	GTGTATAACAACCGACCAGCTC
	FAdV-1-11-R	CATCGAAATACTCGTCTCTGGA
14501–16016	FAdV-1-12-F	GGTAATTACGTGATACCCGACC
	FAdV-1-12-R	CACGTTCCCTCCTTGAAGATC
15976–17000	FAdV-1-13-F	GGTTTACGATTACGAAGATCT
	FAdV-1-13-R	GTCAAAAGAACGGAAGGCATA
16927–17951	FAdV-1-14-F	TGAGATGTTCTTCTGACTATGTGTG
	FAdV-1-14-R	TGAATGAGAGCCTGCAGTTC
17860–18773	FAdV-1-15-F	TTGAAGTTGCAACAGGATCTGG
	FAdV-1-15-R	ACACGTTGGTCATCTGTCCCA
18674–20090	FAdV-1-16-F	ACGGCTTATAATCCCCTTGC
	FAdV-1-16-R	TATTGTGGTCCATGGGCATG
19982–21415	FAdV-1-17-F	AACATGATTCTGCAGTCCAGC
	FAdV-1-17-R	TACTTGACTCTGTATAACTCCCACAG
21354–22784	FAdV-1-18-F	TACATGTTTGACCCTTTCGGG
	FAdV-1-18-R	TTCCTGGCAGCCGTTATCTA
22651–24033	FAdV-1-19-F	ATGTCCACACCGTGCATGC
	FAdV-1-19-R	TTCTGAGCTTCGCTTTCATCG
23949–25391	FAdV-1-20-F	AACCTTCCGAAATGGACCG
	FAdV-1-20-R	ACATACCCATAGCCGTTTGC
25281–26816	FAdV-1-21-F	TGAACAACTGCATCATGTCCAA
	FAdV-1-21-R	AGCATAGTAGCCTATGACAGTAGCG
26672–28138	FAdV-1-22-F	AGGTAAATATCGCAGCTGGG
	FAdV-1-22-R	GTTCATGATGTCGAACCCTAGAA
28038–29537	FAdV-1-23-F	TCAGCCAGAATACAAAACCG
	FAdV-1-23-R	TCAAGGGTTCCATCGTGTTT
29444–30916	FAdV-1-24-F	CTTACAGTCACGAGTAACTCTCTCG
	FAdV-1-24-R	TCAACGACCAGGGTTTTGG
30763–32230	FAdV-1-25-F	AATCTAACGGCAACGCGAT
	FAdV-1-25-R	GTTACCTTTGCATCGTATGGG
32076–33450	FAdV-1-26-F	CTGGAGAGAAATCGCTAAACC
	FAdV-1-26-R	ATGAAGTACGACTTGGAACCG
33342–34827	FAdV-1-27-F	GGAGAGCCATCTTTACGGATAT
	FAdV-1-27-R	AGGCTCCCATTGTTTTATATGC
34711–36190	FAdV-1-28-F	TCACTTCCTGTGTCGTCATTG
	FAdV-1-28-R	AAGCTCGGAAATGGAGGC
36083–37560	FAdV-1-29-F	ATCCCATCTGGAATCCGAC
	FAdV-1-29-R	TCGTAGTCTGCGAAGATTGC
37424–38907	FAdV-1-30-F	AATCCCACCACTAGCCTTAGAG
	FAdV-1-30-R	TCTACCACCACGGGAAAGTC
38808–40311	FAdV-1-31-F	GCCACAATGTAATTACCCACTG
	FAdV-1-31-R	GATGGTTAATGCCTTGTGACG
40224–41696	FAdV-1-32-F	CTGGATACGGCAAACAATACC
	FAdV-1-32-R	TGGCAATTGTTCTAGAAATAGTTTT
41571–42798	FAdV-1-33-F	GGATTTTCCAATGCGTCATT
	FAdV-1-33-R	AGAATGTTCCGAGAAGCTTTTC
42551–43739	FAdV-1-34-F	TAAGTATGGACACCGCCGA
	FAdV-1-34-R	GATGATGTATAATAACCTCAAAAACTAAC

Since JS2017 is nonpathogenic, it may provide an effective viral vector for future poultry vaccine research.

### Data availability.

The nearly complete genome sequence of serotype 1 fowl adenovirus strain JS2017 has been deposited in GenBank under accession no. MK050972.
